# Case Report: CT-guided biopsy of a mediastinal mass in the visceral compartment

**DOI:** 10.3389/fonc.2026.1755977

**Published:** 2026-02-05

**Authors:** Xiangrui Chen, Min Hu, Chengluo Hao, Yunwei Han, Jingting Zhao

**Affiliations:** 1 Department of Oncology, Zigong Third People’s Hospital, Zigong, Sichuan, China; 2 Department of Dermatology, Zigong Third People’s Hospital, Zigong, Sichuan, China; 3 The Department of Oncology, Affiliated Traditional Chinese Medicine Hospital of Southwest Medical University, Luzhou, Sichuan, China; 4 Quality Management Office, Zigong Third People’s Hospital, Zigong, Sichuan, China

**Keywords:** biopsy, CT-guided, mediastinal neoplasms, precision medicine, preprocedural planning

## Abstract

**Background:**

The pathological diagnosis of mediastinal lesions is crucial for precision oncology. While endobronchial ultrasound-guided transbronchial needle aspiration (EBUS-TBNA) is the preferred minimally invasive method for visceral mediastinal lesions, its diagnostic yield can be limited for high-risk lesions surrounded by great vessels. This case demonstrates that under such complex anatomical constraints, a meticulously planned CT-guided percutaneous biopsy serves as a safe and effective alternative.

**Case presentation:**

A middle-aged male patient was highly suspected of having lung cancer with mediastinal metastasis based on clinical and radiological findings. Two successive bronchoscopic biopsies of the pulmonary lesion failed to yield a diagnosis. Although the medical team recommended EBUS-TBNA at a tertiary hospital, the patient opted for a CT-guided biopsy at our institution after considering personal convenience and economic factors. The target was a high-risk mediastinal lymph node located within the vascular “core area” between the aorta and superior vena cava.

**Intervention and outcome:**

Preprocedural planning with contrast-enhanced CT simulated three potential trajectories (transcostochondral, transsternal, transpulmonary). The transsternal approach was prioritized to avoid lung parenchyma, thereby eliminating the risk of pneumothorax—a critical consideration given the patient’s comorbid emphysema and bullae. The initial transcostochondral approach was abandoned due to pain upon vascular contact and restricted maneuverability. The subsequent transsternal approach was successfully performed using a coaxial biopsy system to navigate the narrow vascular space, followed by tract embolization upon needle withdrawal. The procedure was safe, with only minimal, self-resolving mediastinal emphysema. Adequate tissue cores were obtained, enabling a definitive diagnosis of metastatic lung adenocarcinoma.

**Conclusion:**

For complex mediastinal lesions where standard approaches are unsuitable or declined by the patient, a meticulously planned CT-guided percutaneous biopsy based on three-dimensional anatomical assessment is a feasible and valuable diagnostic strategy.

## 1 Introduction

The pathological diagnosis of mediastinal lesions is the cornerstone of precise diagnosis and treatment of tumors. For lesions located in the visceral mediastinum (middle mediastinum), endobronchial ultrasound-guided transbronchial needle aspiration (EBUS-TBNA) has become one of the preferred diagnostic methods due to its minimally invasive nature and the advantage of real-time ultrasound guidance (1–3). However, when lesions are closely surrounded by major blood vessels or separated from the airway by critical structures such as vessels, the specimen yield from EBUS-TBNA may be limited, or there might be blind spots in the puncture path, compromising diagnostic accuracy.

In such complex scenarios, CT-guided percutaneous needle biopsy provides another crucial diagnostic pathway. This technique can clearly delineate the three-dimensional relationship between the lesion and surrounding vessels and organs through CT imaging, thereby enabling the planning of a “safe trajectory” that avoids critical structures (4). Particularly for lesions in certain locations, meticulous path planning (e.g., parasternal, paravertebral approaches) under CT guidance can potentially allow more direct sampling of the lesion while effectively avoiding complications commonly associated with transpulmonary routes, such as pneumothorax and bleeding (5). However, when standard approaches like EBUS-TBNA are declined by patients due to personal or practical considerations (e.g., transfer inconvenience or economic burden), CT-guided biopsy can serve as a carefully evaluated alternative under specific anatomical constraints (6).

This article reports the diagnostic process of a visceral mediastinal mass. Although EBUS-TBNA was a feasible option for the lesion at this site, the patient, after thorough consideration, ultimately underwent CT-guided percutaneous biopsy. Through this case, we explore how meticulous pre-procedural CT evaluation and individualized trajectory planning can establish CT-guided biopsy as a safe and effective diagnostic tool in such complex anatomical settings, with a focus on the rationale for strategy selection and its clinical decision-making value.

## 2 Case description

**Figure 1:**
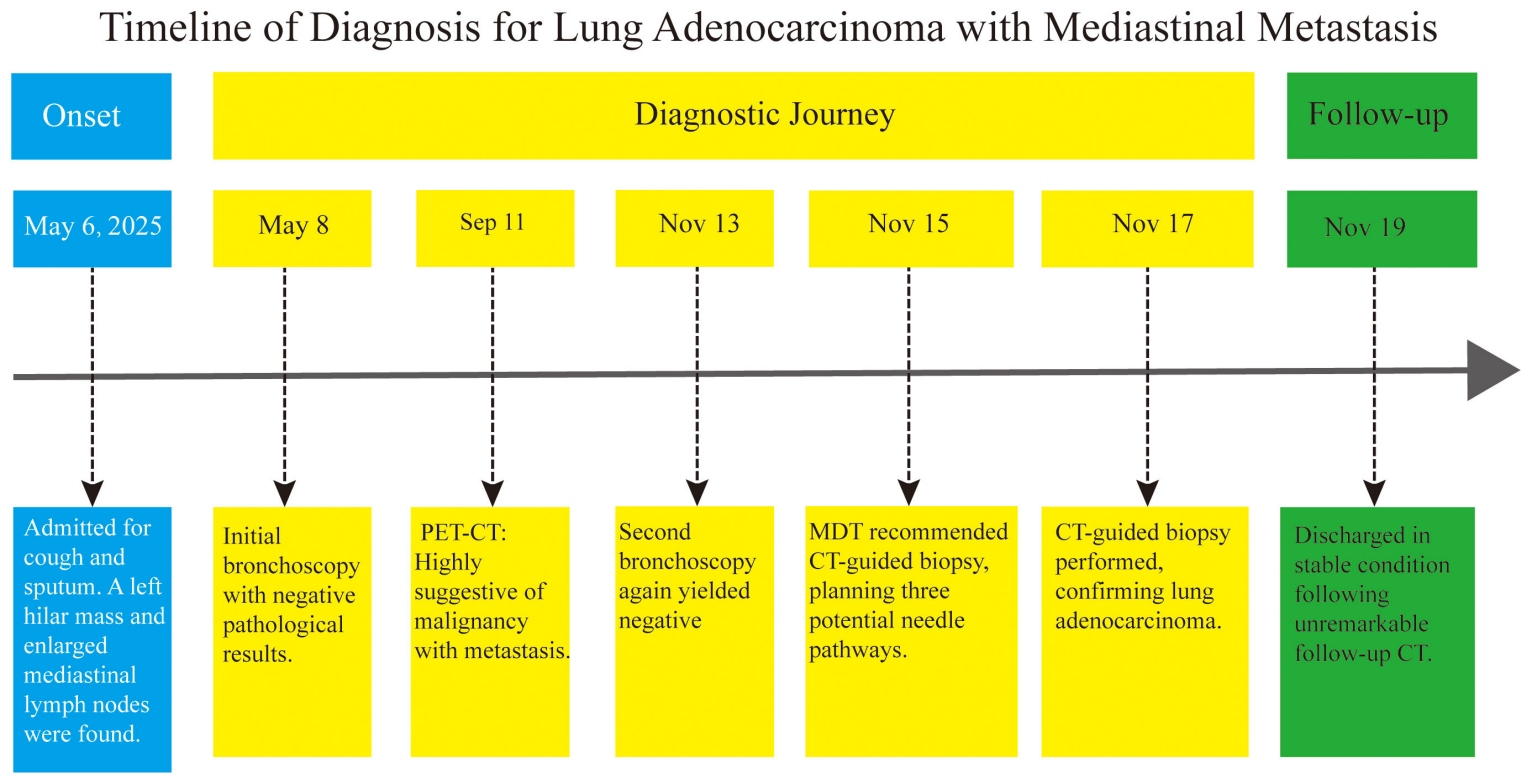
Diagnostic timeline for lung adenocarcinoma with mediastinal metastasis. This timeline summarizes the key diagnostic events in a patient with a lung mass and mediastinal lymphadenopathy. After two negative bronchoscopic biopsies created a diagnostic dilemma, a multidisciplinary team recommended CT-guided biopsy. MDT, multidisciplinary team; PET-CT, positron emission tomography-computed tomography.

We report the case of a middle-aged male patient admitted for evaluation under the strong clinical suspicion of lung malignancy with mediastinal metastasis. Following an episode of pneumonia, a positron emission tomography-computed tomography (PET-CT) scan was highly suggestive of malignancy; however, the initial bronchoscopic biopsy yielded negative results. This report details the successfully and safely performed, carefully planned CT-guided percutaneous biopsy of the mediastinal mass, which secured the pathological diagnosis, with the diagnostic timeline summarized in **Figure 1**. Written informed consent was obtained from the patient for the publication of this case report and any accompanying images.

### 2.1 Patient information

**Figure 2:**
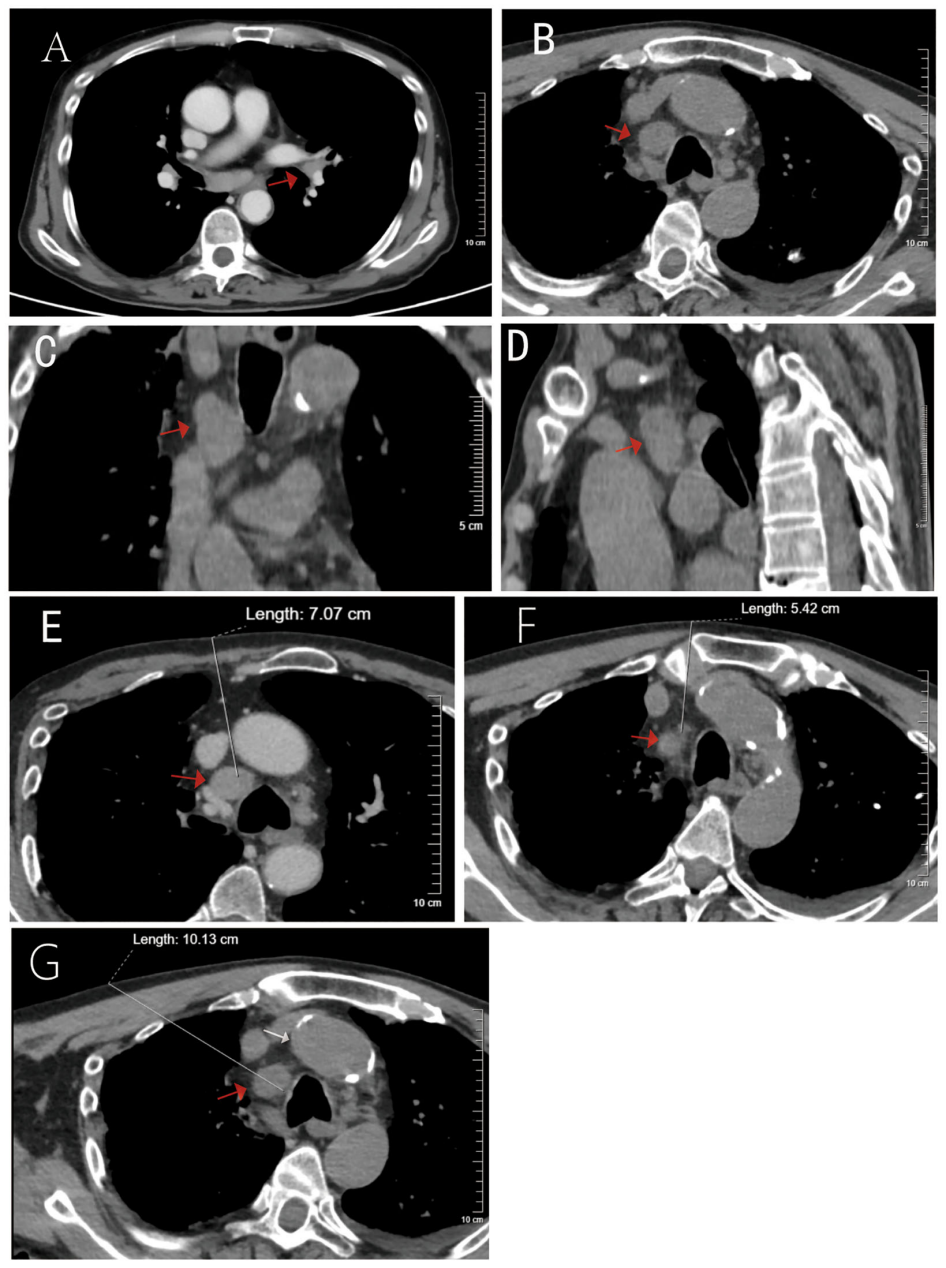
Imaging characteristics of the mediastinal mass and schematic diagram of three preprocedural planned biopsy pathways. **(A–D)** Chest CT images at the initial admission (red arrow indicates the lesion). **(A)** shows a nodule adjacent to the left upper lobe. **(B)** shows enlarged mediastinal lymph nodes. **(C)** Coronal and **(D)** sagittal reconstructions further demonstrate the spatial relationship of the lesion to adjacent structures. **(E–G)** Schematic diagrams of the three planned CT−guided percutaneous biopsy pathways. **(E)** Path 1 (trans−costal cartilage): attempting needle insertion through the space between the aorta and the superior vena cava. **(F)** Path 2 (trans−sternum): planned via the space among the left brachiocephalic vein, right brachiocephalic vein, and the aorta. **(G)** Path 3 (trans−pulmonary): a pathway crossing the lung parenchyma.

A 61-year-old male with a 40-pack-year smoking history (approximately one pack per day for 40 years) and chronic heavy alcohol consumption (100 g of baijiu daily for 40 years) was first admitted to the Department of Respiratory Medicine on May 6, 2025, presenting with cough and fever. The initial chest CT revealed bilateral interstitial pneumonitis, chronic infection, emphysema, pulmonary bullae, a juxtamediastinal nodule in the lingular segment of the left upper lobe (nature to be determined), and lymphadenopathy in the mediastinum and left pulmonary hilum (**Figures 2A-D**). The first fiberoptic bronchoscopy was performed on May 8, 2025, involving forceps biopsy and cryobiopsy of the lingular lobe; pathological examination identified no tumor cells. The patient was diagnosed with “bilateral pneumonia” and was discharged after improvement with symptomatic treatment, including antibiotics.

Following discharge, the patient experienced intermittent coughing, which persisted until September 2025. To further characterize the pulmonary nodule, a PET-CT scan was performed on September 11, 2025. The results were highly suggestive of malignancy: 1. A solid density shadow with increased glucose metabolism adjacent to the nodule near the lingular segmental bronchus of the left upper lobe, suggesting potential lung malignancy; and 2. Multiple enlarged mediastinal lymph nodes with increased metabolism, suggestive of metastasis. The scan also confirmed underlying conditions including chronic inflammation and emphysema.

In November 2025, the patient was readmitted due to cough with white, sticky sputum. Vital signs were stable on admission, and lung auscultation was unremarkable. Laboratory tests showed a significantly elevated carcinoembryonic antigen level of 44.50 ng/mL. A repeat contrast-enhanced chest and abdominal CT confirmed the persistence of the juxtamediastinal nodule in the lingular segment and mediastinal lymphadenopathy. To obtain a pathological diagnosis, a second fiberoptic bronchoscopy with forceps biopsy and cryobiopsy of the lingular lobe was performed on November 13, 2025. The pathology result again reported “chronic mucosal inflammation of the left lingular lobe, no malignant cells identified.” Thus, two attempts via the bronchoscopic route failed to yield a definitive diagnosis.

In summary, the clinical presentation, imaging, and serological findings strongly indicated left upper lobe lung malignancy with mediastinal lymph node metastasis. However, the diagnosis remained elusive due to two negative bronchoscopic biopsies. A multidisciplinary discussion was held to guide further pathological diagnosis. The patient was referred to a higher-level hospital for EBUS-TBNA, with alternatives including thoracoscopic biopsy or CT-guided percutaneous biopsy. Considering the inconveniences and economic burden associated with transfer, the patient opted to be transferred to the Department of Oncology at our hospital for a CT-guided percutaneous biopsy.

### 2.2 Preprocedural trajectory planning

After the patient was transferred to the Department of Oncology and preprocedural evaluations were completed with no contraindications identified, three interventional oncologists held a discussion based on the contrast-enhanced CT scan. They meticulously assessed all potential biopsy targets, with a focus on planning three possible needle trajectories. After evaluation, the largest lesion in the visceral mediastinum (approximately 2.5 × 1.8 × 1.4 cm) was selected as the target, as it offered the highest likelihood of successful sampling. However, this lesion was surrounded by the aorta, superior vena cava, right pulmonary artery, and trachea. The primary lesion in the left hilum was deemed to have no safe access route due to encasement by the left pulmonary artery and veins.

Trajectory 1 (Transcostochondral, **Figure 2E**): A percutaneous approach passing through a costal cartilage into the mediastinum, attempting to reach the target lesion via the space between the aorta and the superior vena cava.Trajectory 2 (Transsternal, **Figure 2F**): A percutaneous approach passing through the sternum into the mediastinum, attempting to access the target via the space between the left brachiocephalic vein, right brachiocephalic vein, and aorta.Trajectory 3 (Transpulmonary, **Figure 2G**): A percutaneous approach passing through the chest wall and lung tissue to enter the mediastinum and reach the target lesion.

To maximize the success rate of tissue acquisition while minimizing the risk of complications, it was decided to prioritize Trajectory 1. Trajectory 2 would be attempted if the first failed, with Trajectory 3 as the last resort. This decision was based on the consideration that the transpulmonary route could increase the risks of pneumothorax, hemoptysis, intrathoracic hemorrhage, and even air embolism, and was therefore not the preferred option. A coaxial biopsy system with a blunt-tip needle technique would be employed during the procedure to minimize potential vascular injury.

### 2.3 Procedural details

**Figure 3:**
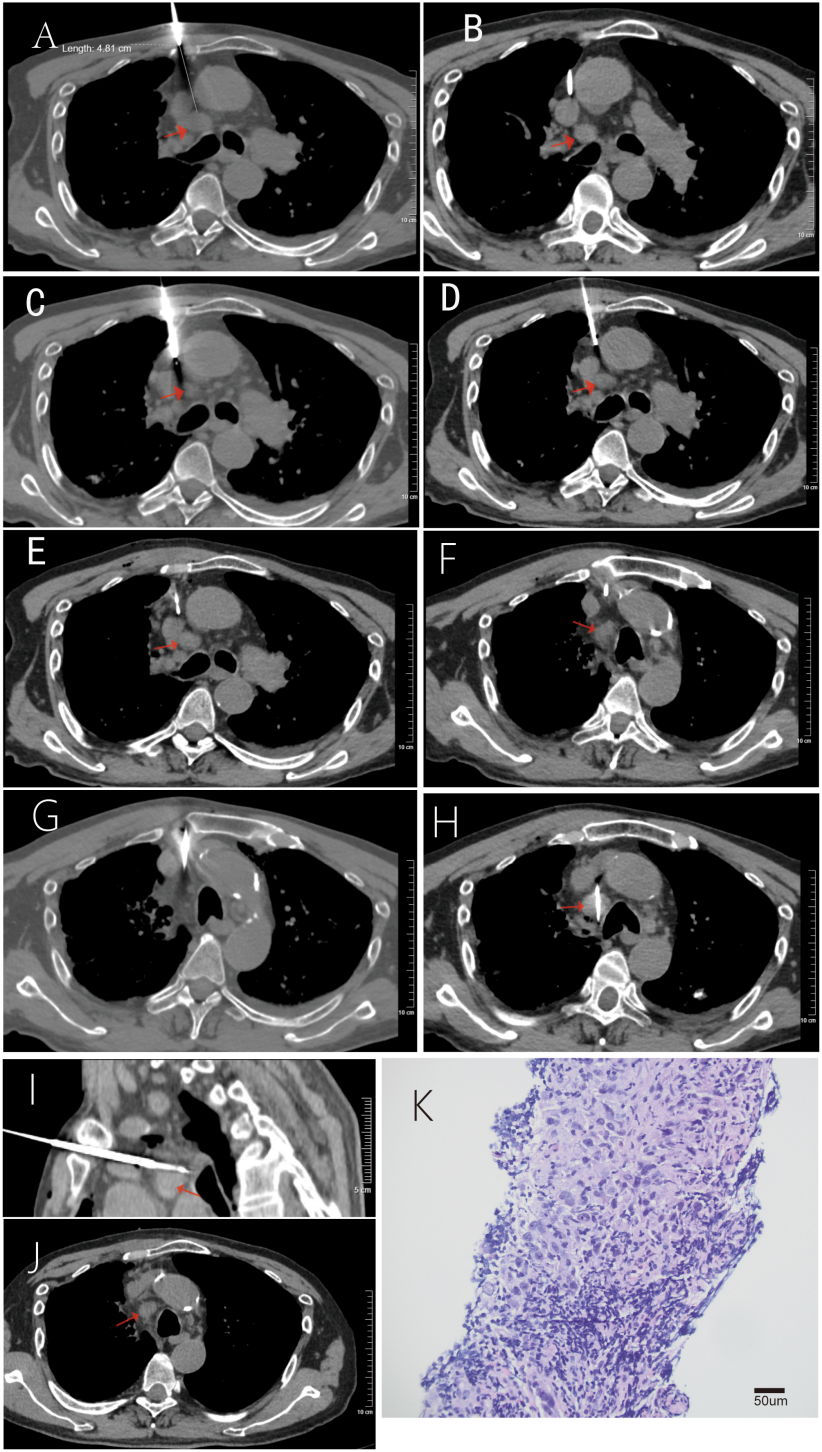
CT-guided biopsy procedure and pathological confirmation. **(A–E)** Failed attempts via Path 1 (red arrow indicates the target lesion; thick, linear high-density opacity represents the biopsy needle). Intraprocedural CT scans show repeated attempts with a 17-gauge coaxial blunt-tip needle to pass through the narrow space between the aorta and the superior vena cava. The procedure was aborted each time because contact of the needle with the vessels caused intolerable pain for the patient. In **(B, E)**, the blunt-tip needle contacts the superior vena cava; in **(C, D)**, it contacts the aorta. **(F–I)** Successful biopsy via Path 2. **(F)** A 13-gauge bone biopsy needle penetrates the sternum. **(G)** A 17-gauge coaxial biopsy needle passes through the space among the left brachiocephalic vein, right brachiocephalic vein, and the aorta. **(H, I)** An 18-gauge semi-automatic biopsy needle samples the target lesion. **(J)** Follow-up CT scan at 48 hours shows complete resolution of minor mediastinal emphysema (arrow) and no active hemorrhage or hematoma. **(K)** Histopathological examination confirmed metastatic lung adenocarcinoma (H&E staining, ×200; inset shows positive TTF-1 immunohistochemical staining).

Intraoperatively, Trajectory 1 was attempted first as planned. After the 17-gauge coaxial introducer needle (TSK Corporation, National Medical Device Import Registration No. 20172140384) penetrated the costal cartilage into the mediastinum, a blunt-tip needle was introduced and attempts were made to navigate the space between the aorta and the superior vena cava. However, multiple attempts were unsuccessful (**Figures 3A–E**). The failure was attributed to two main factors: 1) The patient experienced intolerable, sharp pain prompting immediate cessation of needle advancement each time the blunt tip contacted the vascular wall, even after supplemental injection of 1% lidocaine for local anesthesia around the vessel surface; and 2) The overlying costal cartilage significantly restricted the available angles and range of needle adjustment.

Subsequently, Trajectory 2 was initiated (**Figures 3F–I**). First, a 13-gauge bone biopsy needle was used to carefully penetrate the sternum along the predetermined path. The 17-gauge coaxial biopsy system with the blunt-tip needle was then introduced into the mediastinum. After two attempts, the needle successfully passed through the space between the left brachiocephalic vein, right brachiocephalic vein, and aorta. The operator noted a distinct loss-of-resistance sensation as the needle tip entered the target area, reaching the edge of the lesion. Finally, an 18-gauge semi-automatic biopsy gun was advanced through the coaxial sheath to obtain three core tissue specimens. Following biopsy, while the needle system was slowly withdrawn, a mixture of gelatin sponge particles and hemocoagulase agkistrodon was injected along the needle tract for embolization to prevent local bleeding. An immediate post-procedural CT scan revealed a minimal amount of mediastinal emphysema, with no evidence of active bleeding along the tract or perilesional hematoma.

### 2.4 Follow-up

The patient’s postoperative course was uneventful. The patient reported that aside from the transient pain experienced during needle advancement in the procedure, he had no postoperative discomfort. He denied any chest pain or shortness of breath. A follow-up chest CT scan two days after the procedure showed resolution of the mediastinal emphysema (**Figure 3J**), with no evidence of hemorrhage along the needle tract or perilesional hematoma.Pathological diagnosis confirmed the presence of malignant tumor cells in the mediastinal lymph node. Immunohistochemical staining showed the tumor cells were positive for PCK, CK7, TTF-1, and Napsin A, while negative for p40 and CK5/6. These findings support the diagnosis of metastatic lung adenocarcinoma (**Figure 3K**).The final diagnosis was adenocarcinoma of the left pulmonary hilum with mediastinal lymph node metastasis (cT2N3M0, Stage IIIB). The patient has been discharged and is currently awaiting genetic testing results to guide subsequent treatment.

## 3 Discussion

The pathological diagnosis of mediastinal lesions is fundamental to the accurate staging of lung cancer. For the visceral mediastinal lesion in this case, EBUS-TBNA is recommended as the preferred minimally invasive diagnostic method by several guidelines, owing to its high technical safety (real-time ultrasound allowing for vessel avoidance) and high diagnostic accuracy (7). However, clinical decision-making is a complex process that integrates medical evidence, technical availability, and patient preferences and values. The decision to proceed with CT-guided biopsy was primarily driven by the patient’s preference after weighing personal convenience and economic factors, despite the medical team’s recommendation for EBUS-TBNA at a tertiary center. This decision underscores that in clinical practice, respecting patient autonomy is as crucial as providing evidence-based medical recommendations. Therefore, the focus of this case report is not to compare the superiority of techniques, but rather to explore how to maximize the safety and efficacy of a technique often considered “alternative” or more challenging (CT-guided biopsy) while respecting the patient’s choice. This single-case report demonstrates feasibility but cannot establish generalizability. Its value lies in illustrating meticulous planning principles for highly selected scenarios where standard approaches are declined.

When EBUS-TBNA is not feasible as the primary option, CT-guided percutaneous biopsy becomes a critical technique for obtaining a pathological diagnosis. The unique challenge in this case was that the target lesion was entirely located within the “core area” formed by major vessels such as the aorta and superior vena cava, rendering a conventional transpulmonary approach unacceptably high-risk. Under such circumstances, the success of the procedure hinged entirely on meticulous, beyond-standard preprocedural trajectory planning. We deliberately avoided the transpulmonary route—and its associated risk of pneumothorax—and instead carefully evaluated and successfully executed a retrosternal approach. The key advantage of this chosen trajectory was its complete avoidance of lung parenchyma, thereby fundamentally eliminating the risk of pneumothorax, a consideration of paramount importance for this patient with pre-existing emphysema and bullae. This case demonstrates that for select complex mediastinal lesions with specific anatomical constraints, CT-guided biopsy can be optimized through individualized three-dimensional planning.

The success of this case hinged on transforming the biopsy procedure from an “experience-based maneuver” into a methodically planned intervention guided by imaging. The key technical strategies were twofold. First, the preprocedural multi-trajectory simulation and contingency planning: we systematically planned three distinct trajectories with a clear order of priority. When the primary path failed due to vascular obstruction and patient intolerance to pain, this planning allowed for a rapid and orderly transition to the alternative retrosternal approach, avoiding blind attempts. Second, the intraoperative precision safety techniques, including the use of a coaxial needle biopsy system and a blunt-tip needle to better deflect vessels while navigating narrow spaces (8), and the critical step of tract embolization using a combination of gelatin sponge and hemocoagulase upon needle withdrawal to prevent delayed bleeding from the puncture site (9). The postoperative imaging, showing only minimal mediastinal emphysema and no active hemorrhage, confirmed the safety of this technical strategy. Ultimately, the technique successfully yielded adequate tissue cores, which not only sufficed for conventional pathological diagnosis but also enabled subsequent immunohistochemical subtyping and genetic testing, supporting comprehensive tumor characterization. It must be emphasized that this success relied on the interventional team’s specialized expertise in high-risk mediastinal biopsies, including sternal penetration and navigation within narrow vascular spaces—a level of proficiency not readily replicable in low-volume centers.

Advanced medical decision-making should be a shared journey between the physician and the patient. The physician is responsible for providing the most professional medical information (such as the advantages of EBUS-TBNA), while the patient’s values and preferences (such as an inclination toward CT-guided biopsy) equally deserve respect. Within this framework, the value of the medical team lies in its ability to safely and effectively implement a non-preferred option, when chosen, through technical proficiency and meticulous planning. While this case illustrates a successful approach for a highly specific anatomical scenario, it serves primarily as an educational example for centers with comparable interventional expertise, rather than as evidence to alter clinical pathways. The successful execution of the CT-guided retrosternal biopsy in this case provides a practical example for specific patients with mediastinal lesions who, for various reasons, cannot undergo EBUS-TBNA. In the future, with advancements in image-guidance technology and biopsy instruments, the indications for percutaneous mediastinal biopsy are expected to expand further. However, regardless of technological progress, patient-centered shared decision-making and safety-first procedural planning will remain the enduring themes.

## 4 Conclusion

For complex visceral mediastinal lesions that are challenging to diagnose using conventional minimally invasive methods, this case demonstrates that CT-guided percutaneous biopsy, facilitated by meticulous preprocedural planning and technical optimization, serves as a safe and effective diagnostic tool. This technique successfully circumvented critical vascular structures and provided the crucial pathological basis for subsequent precision therapy, offering an invaluable practical reference for biopsy decision-making in similar high-risk anatomical locations.

## Data Availability

The original contributions presented in the study are included in the article/supplementary material. Further inquiries can be directed to the corresponding author.
